# Graph partitioning MapReduce-based algorithms for counting triangles in large-scale graphs

**DOI:** 10.1038/s41598-022-25243-w

**Published:** 2023-01-04

**Authors:** Ahmed Sharafeldeen, Mohammed Alrahmawy, Samir Elmougy

**Affiliations:** Department of Computer Science, Faculty of Computers and Information, Mansoura University, Mansoura, Egypt

**Keywords:** Computer science, Information technology

## Abstract

Counting number of triangles in the graph is considered a major task in many large-scale graph analytics problems such as clustering coefficient, transitivity ratio, trusses, etc. In recent years, MapReduce becomes one of the most popular and powerful frameworks for analyzing large-scale graphs in clusters of machines. In this paper, we propose two new MapReduce algorithms based on graph partitioning. The two algorithms avoid the problem of duplicate counting triangles that other algorithms suffer from. The experimental results show a high efficiency of the two algorithms in comparison with an existing algorithm, overcoming it in the execution time performance, especially in very large-scale graphs.

## Introduction

Over the last decade, the size of graphs used in social networks has grown significantly due to the increase in data used in these networks. One of the most important problem in social networks is to analyze their large graphs to extract useful information. Sequential algorithms can’t deal with large graphs due to limitations in memory and processing capabilities. We can overcome those problems by applying parallel computing in analyzing these networks. One of the most popular parallel computing methods is MapReduce^1^ which is the-state-of-the-art for processing large scale graphs and is implemented on a cluster of machines using Hadoop^2^ which is an open source framework provided by Apache.

One of the most major problems in graph analysis is to count the number of triangles in a graph, which is called *triangle counting*. Triangle counting is considered the core in many graph analytic operations such as measuring clustering coefficient^3^, transitivity ratio^4^, triangular connectivity, k-truss^5^, etc. Also, there are many real-world applications based mainly on triangle counting such as spam detection, Facebook, and LinkedIn^6^.

In this paper, we propose two new MapReduce algorithms to count triangles in large scale graphs. Our algorithms partition a large graph into sub-graphs, then count triangles in each sub-graph. After partitioning the graph, every triangle in the graph is classified into one of three categories according to the number of the partitioned sub-graphs containing that triangle. The three triangle categories are named *Type-1*, *Type-2*, and *Type-3* triangle.

We evaluate our two MapReduce algorithms locally in a single node running Hadoop, and also distributed in a cluster of 15 nodes running Hadoop. Experimental results show that our two algorithms have better performance in execution time than the existing algorithm, especially for very large-scale graphs.

### Paper organization

Section “Related works“ discusses previous work related to triangle counting. Section “MapReduce” describes MapReduce framework. Section “Triangles count” discusses the triangle counting problem. Section “Our proposed algorithm” presents our proposed algorithms. Section “Experimental results” shows the experimental results, and conclusions are described in “Conclusion” section .

## Related works

There is a lot of research work for counting the number of triangles in a large-scale graph. Cohen algorithm^7^ was the first MapReduce algorithm for counting triangles in a graph. In Cohen algorithm, all 2-path edges; e.g., $\left( u,v\right)$, and $\left( u,w\right)$; are detected first, based on ordering of nodes (i.e. degree of nodes); then, a search is made for edges that connect to this 2-path to form a triangle, e.g., $\left( v,w\right)$. The limitation of this algorithm is to cause network overload and increase computation time. In Suri et al.^8^, a discussion of sequential algorithms (i.e., NodeIterator, and NodeIterator++) for counting triangles is presented; then, they show how to convert these algorithms to MapReduce algorithms. In addition, they developed a MapReduce-based graph partitioning algorithm for counting the number of triangle called GP algorithm. GP algorithm also causes network overload and increases computation time. Moreover, it computes triangles redundantly if two or three nodes of the triangle exist in the same partition. Park et al.^9^ developed an efficient algorithm, called Triangle Type Partition (TTP), for counting triangles in a graph. TTP algorithm enhances the performance of GP algorithm as well as reducing the number of frequently computed triangles. However, TTP algorithm computes triangle redundantly if a triangle three nodes exist in the same partition. In^10^, authors proposed a randomized MapReduce algorithm for counting triangles in a large graph called Colored Triangle Type Partition (CTTP). The idea of CTTP is partitioning the graph at hand into sub-graphs based on randomized coloring function. Another study by Arifuzzaman et al.^11^ proposed a Message Passing Interface (MPI)-based distributed memory parallel algorithm for counting triangles in massive network, called PATRIC. PATRIC algorithm is divided into two phases: Computing balanced load, and Counting triangles. In the first phase, the graph is partitioned into sub-graphs based on the number of processors; so that, computation is balanced between workers in the cluster. In the second phase, each worker counts the triangles in it. After all workers count triangles in their own sub-graph, all counts from workers are merged into a single count by MPI reduce function. Authors used their own modified sequential algorithm, called NodeIteratorN, which is a modified version of NodeIterator++. A novel streaming parallel method, called REPT, to approximately compute the number of triangles in large-scale graphs is proposed by Wang et. al.^12^. Randomly, it divides the graph into several processors, then each processor computes the number of triangles in its sub-graph. Hu et all.^13^ presented a fine-grained (i.e. little size) task distribution method for counting the number of triangles in the graph using GPU. This method overcomes the both of load imbalance and inefficient memory access problems on GPU. In^14^, The authors proposed an algorithm called TRUST, to count the number of triangles using GPU that is based on hashing as well as vertex-centric approach. Another study, proposed by Ghosh et. al.^15^, employed MPI to count the number of triangles in the graph. There is also a few researches in a problem that is similar to the triangle counting problem, called rectangle counting^16–21^.

## MapReduce

MapReduce^1^ is a parallel distributed programming model for processing huge amounts of data (i.e. size is in Terabytes or Petabytes) on large clusters of commodity machines. In this section, we give a brief overview of MapReduce algorithm and how it works.

**Figure 1:**
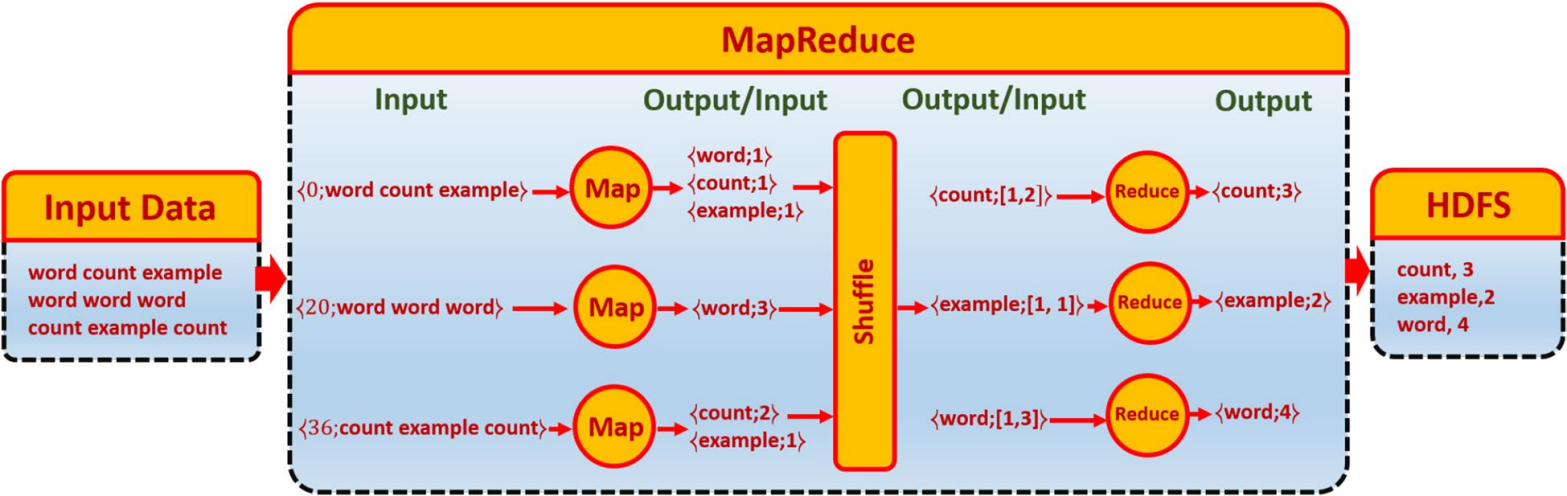
An illustrative example of how MapReduce works.

MapReduce is inspired from Map and Reduce operations in functional programming languages. Using MapReduce, programmer can write a distributed application easily. The most characteristic that MapReduce provides is fault-tolerance, high scalability, and low cost. Hadoop^2^ is an open source framework for implementing a MapReduce on cluster of machines. Hadoop has two major layers: Computation layer (MapReduce), and Storage layer (Hadoop Distributed File System [HDFS]). Inputs and outputs of MapReduce are stored in $\prec key; value\succ$ pairs. MapReduce model consists of three phases: Map, Shuffle, and Reduce. First, Map Phase is written by the programmer. Each Map instance, also called Mapper, receives a line from an input file on HDFS in the form of $\prec key; value\succ$ pairs, where key is start position of line in the file, and value is line content. Output of Map instance is a number of $\prec key; value\succ$ pairs. However, Map instance may have no output if required. The Shuffle Phase is not written by the programmer, as it is done automatically by the framework. The input of the Shuffle phase is the output of Map phase. Shuffle Phase sorts the output of Map phase, and then merge all its elements value; that have the same key as $\prec key;\{value1, value2,\dots \}\succ$. Finally, Reduce Phase is written by the programmer. Each Reduce instance, also called Reducer, receives one of the output pairs $\prec key;\{value1,value2,\dots \}\succ$ of the Shuffle phase as its input. The output of Reduce phase is a number of $\prec key; value\succ$ pairs that are stored on HDFS. An example of how a MapReduce work is shown in Fig. 1.

## Triangles count

List of notations used in this paper is shown in Table 1. Let $G\left( V,E\right)$ be an undirected graph, where *V* is set of vertices, *E* is set of edges, $n=\left| V\right|$, and $m=\left| E\right|$. We define the set of Neighbors of vertex *v* as $\tau \left( v\right) =\{u\in V|\left( v,u\right) \in E\}$, and the degree of vertex *v* as $d\left( v\right) =\left| \tau \left( v\right) \right|$. A triangle, $\Delta \left( u,v,w\right)$, in a graph *G* is any three vertices in the graph which are connected to each other, i.e. $\left( u,v\right) ,\left( v,w\right) ,\left( u,w\right) \in E$. Counting the number of the triangles in the graph is called *triangle counting*. There are many sequential algorithms to count triangles in the graph such as NodeIterator algorithm^8^, NodeIterator++ algorithm^8^, Edge-Iterator algorithm^22^, Forward algorithm^22^, and Compact-Forward algorithm^22^. NodeIterator algorithm is a simple algorithm which identifies neighbors of each vertex, then counts the number of edges among vertex’s neighbors. The running time of NodeIterator is $O\left( \sum _{v\in V} \left( d\left( v\right) \right) ^2 \right)$^8^. While NodeIterator++ algorithm is a modified version of NodeIterator. The problem of NodeIterator is to count triangle six times. When it passes by a vertex, it selects the edge connected to it, so each edge is selected two times as it has two end vertices, so for a triangle the three edges are counted six times. NodeIterator++ avoids this problem by using a total order on all of the vertices denoted by $\succ$, e.g. $u\succ v$ if $d\left( u\right) > d\left( v\right)$. The running time of NodeIterator++ is $O\left( m^{3/2}\right)$^8^. Another triangle counting algorithm is Edge-Iterator algorithm which iterates each edge $\left( u,v\right) \in E$, and computes the neighbors of source vertex *u* and target vertex *v*, then counts the common neighbors of *u* and *v*. Forward algorithm is another sequential algorithm for counting triangles in the graph which is an enhanced version of Edge-Iterator which doesn’t compare all neighbors of two adjacent vertices. The running time of Forward algorithm is $O\left( m^{3/2}\right)$^23^ and its memory space has $\theta \left( 3m+3n\right)$^23^. An enhancement version of Forward algorithm is Compact-Forward algorithm shown in^23^ that reduces memory space from $\theta \left( 3m+3n\right)$ to $\theta \left( 2m+2n\right)$. In another hand, there are many MapReduce algorithms to count triangles in an enormous graph as mentioned in “Related work” section.

**Table 1:** Notations used in this paper.

Symbol	Description
$G\left( V,E\right)$	Undirected graph with $\left| V\right|$ vertices, and $\left| E\right|$ edges.
$\left( u,v\right)$	An edge between *u*, *v*; $\left( u,v\right) \in E$.
*n*	Number of vertices.
*m*	Number of edges.
$\tau \left( u\right)$	Set of neighbors of a node *u*.
$d\left( u\right)$	Number of neighbors of a node *u*.
$\rho$	Number of partitions.
$P\left( u\right)$	Partition number of a node *u*.
$\Delta \left( u,v,w\right)$	Triangle; i.e. $\left( u,v\right) ,\left( u,w\right) ,\left( v,w\right) \in E$.
$G_i=\left( V_i,E_i\right)$	Sub-graph of *G* with $V_i$ vertices; 1-partition.
$G_{ij}=\left( V_{ij},E_{ij}\right)$	Sub-graph of *G* with $V_{ij}=V_{i}\cup V_{j}$, where $i\ne j$; 2-partition.
$G_{ijk}=\left( V_{ijk},E_{ijk} \right)$	Sub-graph of *G* with $V_{ijk}=V_i\cup V_j \cup V_k$, where $i\ne j\ne k$; 3-partition

## Our proposed algorithm

**Figure 2:**
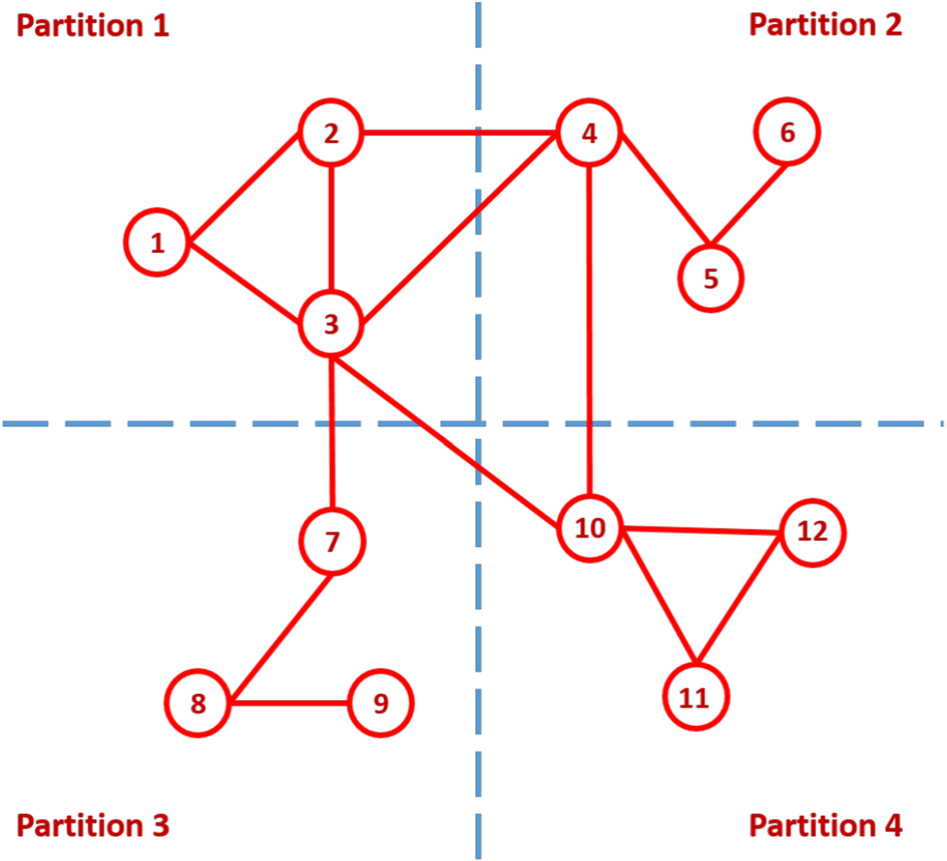
Graph input and partition example, $\rho =4$.

We propose two enhanced MapReduce algorithms to count the number of triangles in large-scale graphs. Those algorithms avoid duplication problem in TTP algorithm. Before, we propose two algorithms, some terms are required to understand those algorithms. In our work, each triangle in the graph $\Delta \left( u,v,w\right)$ can be classified either *Type-1*, *Type-2* or *Type-3* where:

- *Type-1* the three nodes of the triangle are in the same partition, e.g. $\Delta \left( 1,2,3\right)$ shown in Fig. 2.
- *Type-2* two nodes of a triangle are in the same partition, and the third node exists in a different partition, e.g. $\Delta \left( 2,3,4\right)$ shown in Fig. 2.
- *Type-3* each of the three nodes of the triangle exists in different partitions, e.g. $\Delta \left( 3,4,10\right)$ shown in Fig. 2.

**Figure 3:**
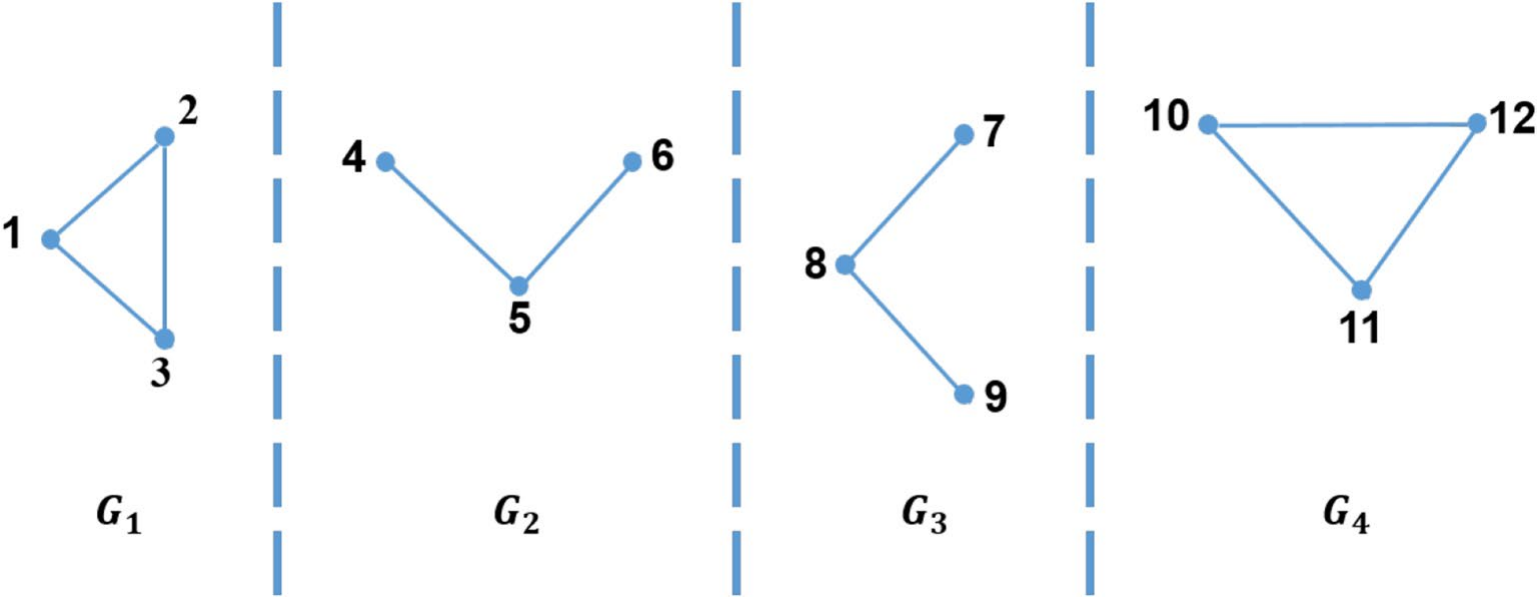
1-partition of the graph in Fig. 2.

**Figure 4:**
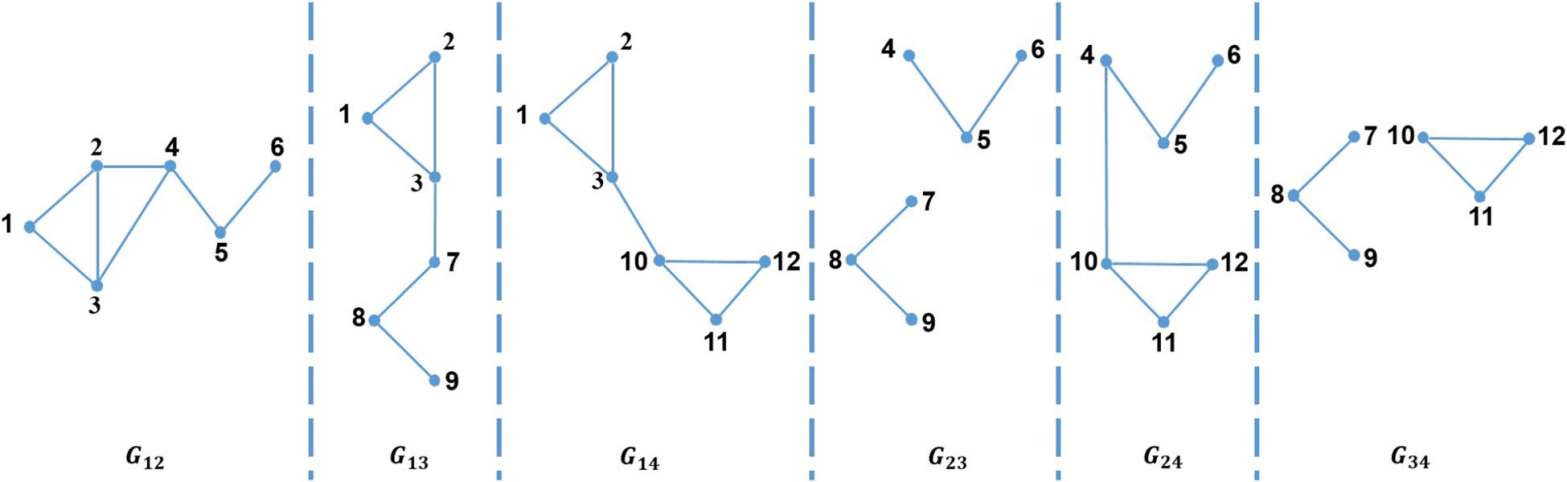
2-partition of the graph in Fig. 2.

**Figure 5:**
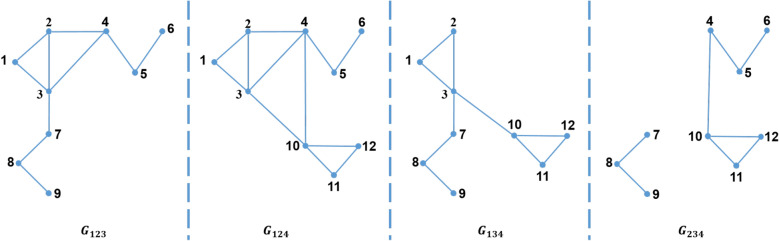
3-partition of the graph in Fig. 2.

Moreover, there are three types of partitioning a graph into a set of sub-graphs which are *1-partition*, *2-partition* or *3-partition*. These three types represent the three types of the triangle that can be defined as follows:

- *1-partition* 1-partition graph is a sub-graph which is denoted by $G_i=\left( V_i,E_i\right)$, where $1 \le i \le \rho$; i.e. $V=\bigcup _{i=1}^\rho V_i$ . For every vertex *v* in this sub-graph, the partition number of this vertex, $P\left( v\right)$, equals *i*. For example, for $\rho =4$, the 1-partition sub-graphs of the graph shown in Fig. 2 are $G_1$, $G_2$, $G_3$, and $G_4$ as shown in Fig. 3. In general, for any graph partitioned in $\rho$ sub-graphs, there are $\rho$ 1-partition sub-graphs.
- *2-partition* 2-partition graph is denoted by $G_{ij}=\left( V_{ij},E_{ij}\right)$ for $1\le i<j\le \rho$. This graph contains every vertex *v* of the graph if the partition number of this vertex, $P\left( v\right)$, equals *i* or *j*. For example, for $\rho =4$, the 2-partition sub-graphs of the graph shown in Fig. 2 are $G_{12}$, $G_{13}$, $G_{14}$, $G_{23}$, $G_{24}$, and $G_{34}$ as shown in Fig. 4. In general, for any graph divided into $\rho$ sub-graphs, there are $$\left( {\begin{array}{c}\rho \\ 2\end{array}}\right) $$ 2-partition sub-graphs.
- *3-partition* 3-partition sub-graph is denoted by $G_{ijk}=\left( V_{ijk},E_{ijk} \right)$ for $1\le i<j<k\le \rho$ which is a sub-graph with partition number of every vertex in such graph, $P\left( v\right)$, equals to *i*, *j* or *k*. For example, for $\rho =4$, the 3-partition sub-graphs of the graph shown in Fig. 2 are $G_{123}$, $G_{124}$, $G_{134}$, and $G_{234}$ as shown in Fig. 5. In general, for any graph separated into $\rho$ sub-graphs, there are $$\left( {\begin{array}{c}\rho \\ 3\end{array}}\right) $$ 3-partition sub-graphs.

### One three partition

Our first proposed MapReduce-based algorithm is *One Three Partition (OTP)*. The algorithm is shown in listing Algorithm 1. OTP algorithm partitions a graph into either **1-partition**, or **3-partition** sub-graphs. Then, it counts the number of triangles in each sub-graph.
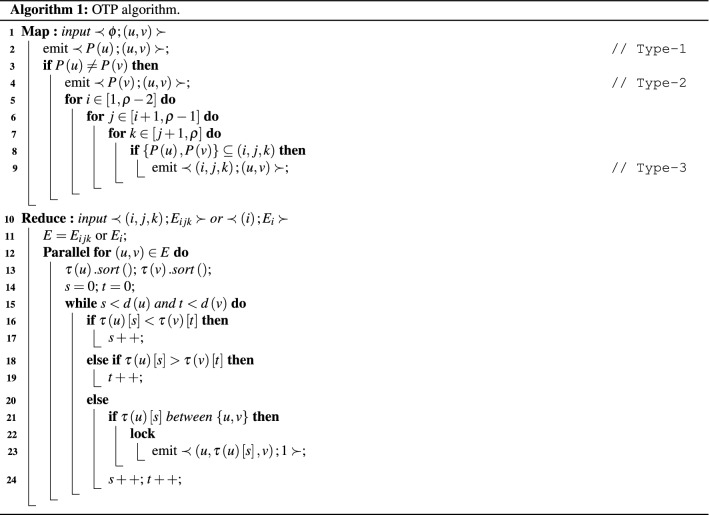


**Figure 6:**
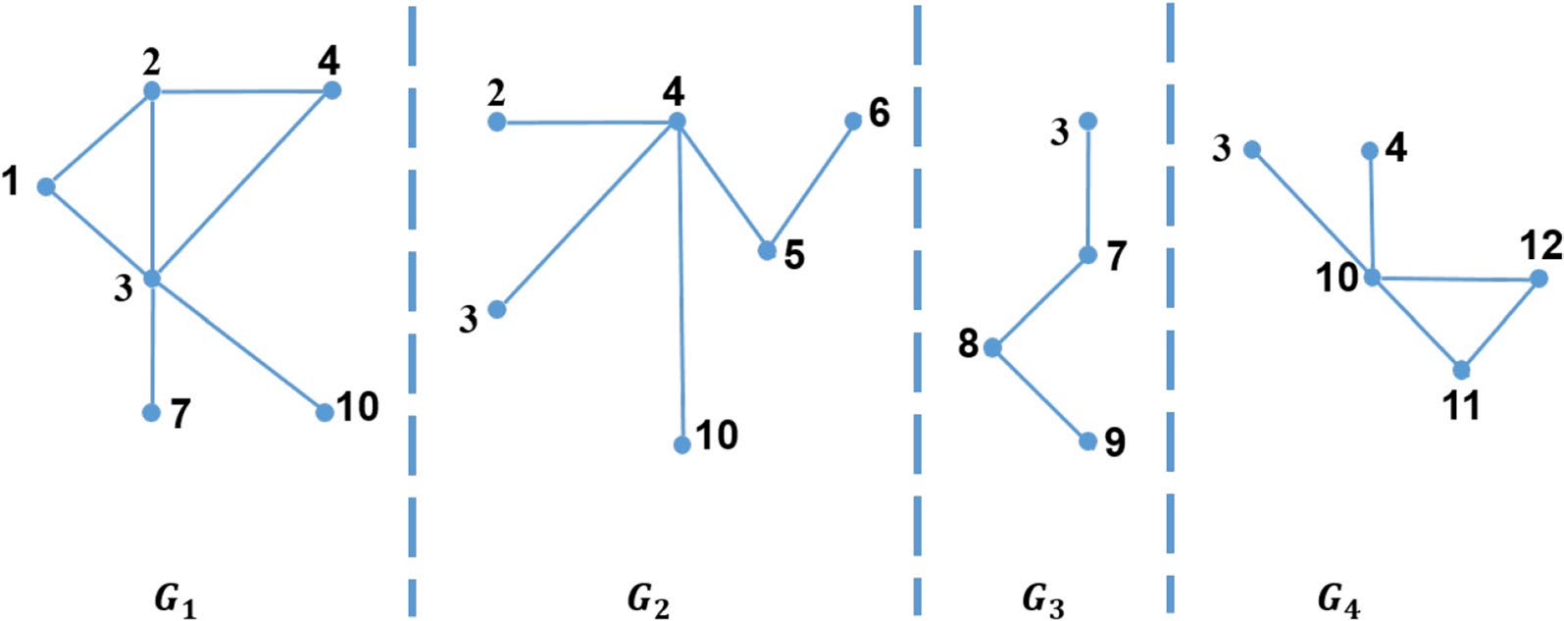
1-partition of the graph in Fig. 2 using OTP.

**Figure 7:**
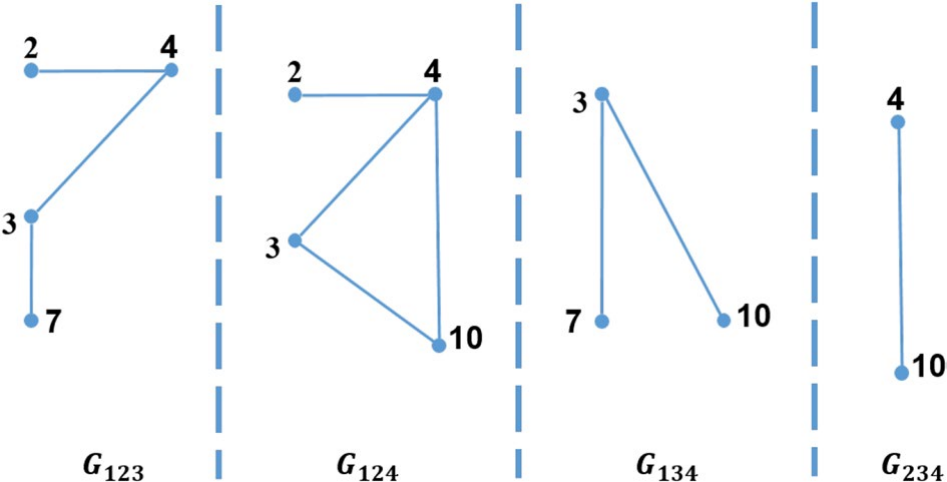
3-partition of the graph in Fig. 2 using OTP.

An edge (*u*, *v*) is called *inner-edge* if both node *u* and *v* exist in the same partition; otherwise is called *cross-edge*. As mentioned before, TTP algorithm processed Type-1 redundantly while Type-2, and Type-3 processed only once. Type-3 only contains cross-edges while Type-2 contain inner-edges and cross-edges, and Type-1 contains only inner-edges. Hence, OTP avoids duplication problem by treating Type-1 and Type-2 at the same time in 1-partition sub-graphs and Type-3 alone in 3-partition sub-graphs. OTP divides graph into $\rho$ equal sized sub-graphs. Since, Type-2 triangle contains one inner-edge and two cross-edges; where each cross-edge is included in two sub-graphs. So, cross-edges are converted to inner-edges by duplicating cross-edges in both two sub-graphs according to partition number of two vertices of those edges. Therefore, OTP algorithm treats Type-2 triangle as Type-1 triangle where both types belong to 1-partition sub-graphs. For example, $\Delta \left( 1,2,3\right)$ is a Type-1 triangle, $P\left( 1\right) =P\left( 2\right) =P\left( 3\right) =1$, so $\left( 1,2\right)$, $\left( 1,3\right)$, and $\left( 2,3\right)$ are only in $G_1$; while $\Delta \left( 2,3,4\right)$ is a Type-2 triangle, where the inner-edge $\left( 2,3\right)$, $P\left( 2\right) =P\left( 3\right) =1$, presents only in $G_1$, and cross-edges $\left( 2,4\right)$ and $\left( 3,4\right)$ [$P\left( 2\right) =P\left( 3\right) =1$ and $P\left( 4\right) =2$] are in both $G_1$ and $G_2$. So, we put each edge of Type-1 and Type-2 to a single sub-graph according to partition number of the vertex and if partition number of two nodes are different, edge is put in two sub-graphs as shown in Fig. 6. 3-partition sub-graphs of OTP algorithm contain only Type-3 triangles (i.e. cross-edges only). For example, $\Delta \left( 3,4,10\right)$ is a Type-3 triangle where the cross edges $\left( 3,4\right)$, $\left( 3,10\right)$, and $\left( 4,10\right)$ present only in $G_{124}$ as shown in Fig. 7.

OTP algorithm consists of Map and Reduce functions. In the Map function (Lines 1–9), a graph is divided into 1-partition and 3-partition sub-graphs. Each edge of the graph is sent to Map instance as input. If edge $\left( u,v\right)$ is inner-edge, the output pair of Map instance is $\prec P\left( u\right) ; \left( u,v\right) \succ$ (Line 2), where $P\left( u\right)$ returns an integer within $\left[ 1,\rho \right]$ that refers to partition number of a node *u*. This mean that the edge $\left( u,v\right)$ is in $G_{P\left( u\right) }$. If edge $\left( u,v\right)$ is a cross-edge, then this edge may be belonging to 1-partition or 3-partition sub-graph as explained earlier. So, we treat it as 1-partition and distribute this edge to the two different 1-partition graphs (Line 2 and Line 4) and also distribute it as a 3-partition sub-graph (Line 9). So, the output of Map instance will be $\prec P\left( u\right) ; \left( u,v\right) \succ$ (Line 2), $\prec P\left( v\right) ; \left( u,v\right) \succ$ (Line 4), and $\prec \left( i,j,k\right) ; \left( u,v\right) \succ$, if $\{P\left( u\right) ,P\left( v\right) \}$ belongs to $G_{ijk}$ for all $1 \le i<j<k\le \rho$ (Line 9). For example, in Fig. 2, if Map instance input is $\left( 2,4\right)$, where $P\left( 2\right) =1$ and $P\left( 4\right) =2$, the output will be $\prec 1; \left( 2,4\right) \succ$, $\prec 2; \left( 2,4\right) \succ$, $\prec \left( 1,2,3\right) ; \left( 2,4\right) \succ$, and $\prec \left( 1,2,4\right) ; \left( 2,4\right) \succ$. The output of Map instance will be as $\prec key; value\succ$, where key refers to the graph number (1-partition or 3-partition) and value refers to the edge belonging to such graph. After all map instances complete, all values of map outputs are combined together if they have the same key as mentioned in “MapReduce” section. In the reduce function (Lines 10–24), triangles are counted and identified in each sub-graph. The input of each reduce instance is a graph number (1-partition or 3-partition) as a key and all edges belonging to this graph as a value. The function in the reduce step is based on Compact-forward algorithm^23^ in which it is parallelized to enhance the performance time of the algorithm. For each edge $\left( u,v\right)$ in the graph, search for a common neighbor *w* for both *u* and *v*; i.e. $w\in \tau \left( u\right)$ and $w\in \tau \left( v\right)$. The output of reduce instance is $\prec \left( u,w,v\right) ; 1\succ$; if a common neighbor *w* is found between *u* and *v* (Lines 21–23) to avoid processing the edges of triangle three times. To avoid the concurrency problem, we use the lock mechanism in Line 22 to avoid race condition problem that may arise when two different iterations write their own result to the same location of the file. For example, in Fig. 2, if the input of reduce instance is $\prec \left( 1,2,4\right) ; \{\left( 2,4\right) , \left( 3,4\right) , \left( 3,10\right) , \left( 4,10\right) \}\succ$, the output will be $\prec \left( 3,4,10\right) ; 1\succ$ only, not $\prec \left( 4,3,10\right) ; 1\succ$ or $\prec \left( 3,10,4\right) ; 1\succ$. In lines 16–19, we search for the next minimum neighbor of two nodes of the edge if this neighbor is not common between source and destination vertices of the edge.

#### Analysis

##### **Lemma 1**

*Each triangle in the graph is counted exactly once by OTP.*

##### *Proof*

Each of Type-1 and Type-2 triangles appears only once in one of the ith 1-partition sub-graph $G_i$. Since, Type-1 $\left( u,w,v\right)$ triangle, i.e. $P\left( u\right) =P\left( w\right) =P\left( v\right)$, appears only in $G_{P\left( u\right) }$, i.e. $u,w,v \in G_{P\left( u\right) }$, and Type-2 $\left( u,w,v\right)$ triangle, i.e. $P\left( u\right) =P\left( w\right)$ and $P\left( w\right) \ne P\left( v\right)$, appears only in $G_{P\left( u\right) }$, i.e. $\left( u,w\right) ,\left( w,v\right) ,\left( u,v\right) \in G_{P\left( u\right) }$ and $\left( w,v\right) ,\left( u,v\right) \in G_{P\left( v\right) }$. Therefore, Type-1 and Type-2 triangles are counted correctly and only once. Each one of Type-3 triangles appears only once in one of the 3-partition sub-graphs. Since, Type-3 $\left( u,w,v\right)$ triangle, i.e. $P\left( u\right) \ne P\left( w\right) \ne P\left( v\right)$, appears only in $G_{P\left( u\right) P\left( v\right) P\left( w\right) }$, where all of $P\left( u\right) ,P\left( v\right) ,P\left( w\right) \in \left[ 1,\rho \right]$; i.e. $\left( u,w\right) ,\left( w,v\right) ,\left( u,v\right) \in G_{P\left( u\right) P\left( v\right) P\left( w\right) }$. Therefore, Type-3 triangles also are counted correctly and only once. Thus, all triangles in the graph are counted exactly once. $\square$

##### **Lemma 2**

*Expected number of all map instances output of OTP is *$m\left( \rho -1+\frac{1}{\rho }\right) =O\left( m\rho \right)$.

##### *Proof*

The proof consists of two consequent steps. In the first step, if a map instance input is an inner-edge, then the output is $G_i$ where $i\in \left[ 1,\rho \right]$ and *i* is the partition number of this edge. Therefore, every inner-edge in the graph appears only in one sub-graph. The probability that an edge is an inner-edge is $\frac{1}{\rho }$. So, the probability of all inner-edge in the graph is $\frac{m}{\rho }$. Therefore, the expect size of inner-edges output is:

1 $$\begin{aligned} \frac{m}{\rho }\times 1=\frac{m}{\rho } \end{aligned}$$

In the second step, if a map instance input is a cross-edge, then the output is both $G_i$ and $G_{ijk}$, where $1\le i<j<k\le \rho$. Since, the output of every cross-edge for $G_i$ is generated two times and the output of every cross-edge for $G_{ijk}$ is $\left( \rho -2\right)$ times. So, the total output of every cross-edge is $2+\left( \rho -2\right) =\rho$ times. Hence, the probability of cross-edge is $\left( 1-\frac{1}{\rho }\right) m=\frac{\rho -1}{\rho } m$. Therefore, the expected number of cross-edges output is:

2 $$\begin{aligned} \frac{\rho -1}{\rho }m\times \rho =\left( \rho -1\right) m \end{aligned}$$

From the above two steps, we include that the expected number of all map instances output of OTP is:

3 $$\begin{aligned} \frac{m}{\rho }+\left( \rho -1\right) m=m\left( \rho -1+\frac{1}{\rho }\right) =O\left( m\rho \right) \end{aligned}$$

$$\square $$

##### **Lemma 3**

*Expected number of each reduce instance input is* $O\left( \frac{m}{\rho ^2}\right)$.

##### *Proof*

Each reduce instance input is either $\prec \left( i\right) ; E_i\succ$ or $\prec \left( i,j,k\right) ; E_{ijk}\succ$. The probability that two nodes of the edge are in a specific partition is $\frac{1}{\rho }\times \frac{1}{\rho }=\frac{1}{\rho ^2}$. For the 1-partition sub-graph, it contains inner-edges and cross-edges of the graph. Since the expected number of two nodes of inner-edges in 1-partition sub-graph equals $\frac{1}{\rho }\times \frac{1}{\rho }=\frac{1}{\rho ^2}$, and the expected number of two nodes of cross-edges in the same partition equals $\frac{1}{\rho }\times \frac{1}{\rho }+\frac{1}{\rho } \times \frac{1}{\rho }=\frac{2}{\rho ^2}$. Hence, for *m* edges, the expected number of two nodes of the inner-edges (cross-edges) in the same partition equals $m\times \frac{1}{\rho ^2} =\frac{m}{\rho ^2}$ ($m\times \frac{2}{\rho ^2} =\frac{2m}{\rho ^2}$). Therefore, the expected number of edges in 1-partition is:

4 $$\begin{aligned} \frac{m}{\rho ^2}+\frac{2m}{\rho ^2}=\frac{3m}{\rho ^2}=O\left( \frac{m}{\rho ^2}\right) \end{aligned}$$

For the 3-partition, it contains cross-edge only. The number of two nodes of the edge in 3-partition equals $$\left( {\begin{array}{c}3\\ 2\end{array}}\right) $$; hence, the expected number of edges in 3-partition is:

5 $$\begin{aligned} \left( {\begin{array}{c}3\\ 2\end{array}}\right) \times \frac{m}{\rho ^2}=\frac{3m}{\rho ^2}=O\left( \frac{m}{\rho ^2}\right) \end{aligned}$$

From the above two equations, we include that for any input, reduce instance takes $O\left( \frac{m}{\rho ^2}\right)$. $\square$

##### **Lemma 4**

*The running time of reduce instance of sparse graph is* $O\left( m\right)$.

##### *Proof*

The running time of step 11 is $O\left( m\right)$, the running time of step 12 is $O(\lg m$+ the running time of steps 13-24), the running time of step 13 is $O\left( k \lg k\right)$ (Assume, the number of neighbors is *k*; using Heap Sort Algorithm), the running time of step 14 is $O\left( 1\right)$, and the running time of steps 15-24 is $O\left( k\right)$. Therefore, the running time of reduce instance is:

$$\begin{aligned}{}&m+\lg m+k \lg k+1+k \le m + m + k \lg k+1+k\\&\quad =2m + k \lg k+1+k\\&\quad \le 2m+k \lg k+1+k \lg k, \quad \text {for } k\ge 2\\&\quad = 2m+2k \lg k+1\\&\quad \le 2m+2k \lg k+m, \quad \text {for } m\ge 1\\&\quad =O\left( m+k \lg k\right) \end{aligned}$$

For dense graph, $k=\frac{m}{2}$, then the running time of reduce instance is $O\left( m \lg m\right)$.

For sparse graph, $k<m$, then the running time of reduce instance is $O\left( m\right)$.

From Lemma 3, reduce instance takes $O\left( \frac{m}{\rho ^2}\right)$ as input, and assume that the graph is a sparse graph; Therefore, the running time of reduce instance is $O\left( \frac{m}{\rho ^2}\right)$. $\square$

##### **Theorem 1**

*The running time of reduce instance of OTP algorithm is better than TTP algorithm.*

##### *Proof*

From Lemma 4 (Assume graph is a sparse graph), the running time of OTP algorithm is $O\left( \frac{m}{\rho ^2}\right)$. TTP algorithm also takes $O\left( \frac{m}{\rho ^2}\right)$ as input and the running time of reduce instance is $O\left( m^{3/2}\right)$^9^. Hence, the running time of reduce instance in TTP algorithm is $O\left( \left( \frac{m}{\rho ^2} \right) ^{3/2}\right)$. Therefore, the running time of reduce instance of OTP algorithm is better than TTP algorithm. $\square$

### Enhanced two three partition

Our second proposed MapReduce algorithm is called *Enhanced Two Three Partition (ETTP)*. The algorithm is shown in listing Algorithm 2. The algorithm partitions the graph into number of equal sized sub-graphs in which each sub-graph can be either **2-partition**, or **3-partition** sub-graph. Then, it counts and identifies triangles in each sub-graph.
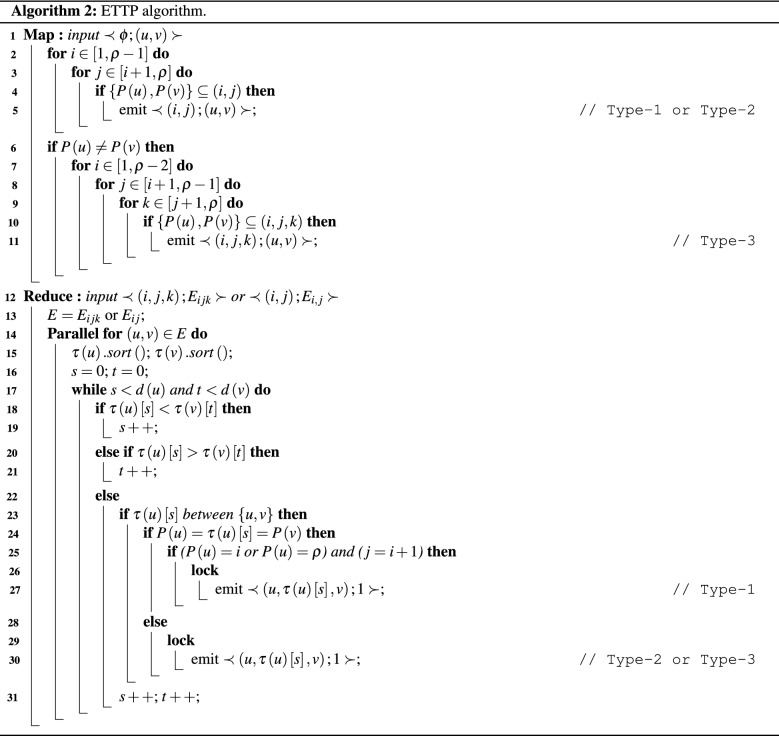


ETTP algorithm is an improved version of TTP algorithm which avoids the duplication problem, that TTP algorithm suffers from, through counting Type-1 only once in the first 2-partition it belongs to, as will be shown later. Since, Type-1 triangles are identified in 1-partition or 2-partition sub-graphs where the three vertices of the triangle belong to. Therefore, ETTP algorithm treats Type-1 (i.e. inner-edges) and Type-2 (i.e. inner-edges and cross-edges) triangles at the same time in the 2-partition sub-graphs while it treats Type-3 triangles alone in 3-partition sub-graphs. In ETTP algorithm, 2-partition sub-graph, $G_{ij}$ for $1\le i<j \le \rho$, contains edges $\left( u,v\right)$ in which partition number of two nodes of those edges equals *i* or *j*; i.e. $\{P\left( u\right) ,P\left( v\right) \}\subseteq G_{ij}$. For example, $\Delta \left( 1,2,3\right)$ is a Type-1 triangle, $P\left( 1\right) =P\left( 2\right) =P\left( 3\right) =1$, so $\left( 1,2\right)$, $\left( 1,3\right)$, and $\left( 2,3\right)$ are in $G_{12}$, $G_{13}$ and $G_{14}$; while $\Delta \left( 2,3,4\right)$ is a Type-2 triangle, where inner-edge $\left( 2,3\right)$, $P\left( 2\right) =P\left( 3\right) =1$, is in $G_{12}$, $G_{13}$ and $G_{14}$, and cross-edges $\left( 2,4\right)$, and $\left( 3,4\right)$ are in $G_{12}$; as shown in Fig. 4. Moreover, 3-partition graph of ETTP, $G_{ijk} 1\le i<j<k\le \rho$, contains only cross-edges in which partition number of two nodes of these edges equals *i*, *j*, or *k*; i.e. $\{P\left( u\right) ,P\left( v\right) \}\subseteq \{i,j,k\}$. For example, $\Delta \left( 3,4,10\right)$ is a Type-3 triangle, where $\left( 3,4\right)$, $\left( 3,10\right)$, and $\left( 4,10\right)$ present only in $G_{124}$ as shown in Fig. 7. Therefore, 3-partition graph of ETTP should be used to count Type-3 triangles of the graph.

ETTP consists of Map and Reduce functions. In the map function (Lines 1–11), a graph is divided into both 2-partition and 3-partition sub-graphs. Each edge of the graph is sent to Map instance as input. If edge $\left( u,v\right)$ is an inner-edge, the output pair of Map instance is $\prec \left( i,j\right) ; \left( u,v\right) \succ$ if $\{P\left( u\right) ,P\left( v\right) \}$ belongs to $G_{ij}$ for all $1\le i<j\le \rho$ (Lines 2–5). For example, in Fig. 2, if Map instance input is $\left( 2,3\right)$, where $P\left( 2\right) =P\left( 3\right) =1$, the output is $\prec \left( 1,2\right) ; \left( 2,3\right) \succ$, $\prec \left( 1,3\right) ; \left( 2,3\right) \succ$ and $\prec \left( 1,4\right) ; \left( 2,3\right) \succ$. If edge $\left( u,v\right)$ is a cross-edge, then this edge may be belonging to 2-partition or 3-partition graph. So, we treat it as 2-partition and distribute this edge to all 2-partition sub-graphs to which it belongs and also distribute it as 3-partition graph. So, the output of Map instance is $\prec \left( i,j\right) ; \left( u,v\right) \succ$ if $\{P\left( u\right) ,P\left( v\right) \}$ belongs to $G_{ij}$ for all $1\le i<j\le \rho$ (Lines 2–5), and $\prec \left( i,j,k\right) ; \left( u,v\right) \succ$ if $\{P\left( u\right) ,P\left( v\right) \}$ belongs to $G_{ijk}$ for all $1\le i<j<k\le \rho$ (Lines 7–11). For example, in Fig. 2, if Map instance input is $\left( 2,4\right)$, where $P\left( 2\right) =1,P\left( 4\right) =2$, the output is $\prec \left( 1,2\right) ; \left( 2,4\right) \succ$, $\prec \left( 1,2,3\right) ; \left( 2,4\right) \succ$ and $\prec \left( 1,2,4\right) ; \left( 2,4\right) \succ$. Thus, the output of Map instance is $\prec key; value\succ$, where key refers to the graph partition number (2-partition or 3-partition) and value refers to the edge belonging to that graph. After all map instances complete, all values of map outputs are aggregated together if they have the same key as mentioned in “MapReduce” section. In the reduce function (Lines 12–31), triangles are counted and identified in each sub-graph. The input of each reduce instance is the graph partition number (2-partition or 3-partition) as a key and all edges belonging to this graph as a value. Reduce instance algorithm of ETTP is also based on Compact-forward algorithm. For each edge $\left( u,v\right)$ in the graph, search for a common neighbor *w* in $\tau \left( u\right)$ and $\tau \left( v\right)$. If *w*’s id is between *u*’s and *v*’s ids (i.e. $w\prec u,v$) (Line 23) and the triangle is a Type-1 triangle (Line 24), then it counts the triangle only once when the partition number of vertex, $P\left( u\right)$, equals to *i* and $\left( j=i+1\right)$ (i.e. the first 2-partition sub-graph belongs to it), or if this triangle exists in the last partition [i.e. $P\left( u\right) =\rho$], then the triangle is counted and identified only in the last sub-graphs [i.e. $P\left( u\right) =\rho$ and $j=i+1$] (Lines 25–27). So, Lines 24–27 of the algorithm count Type-1 triangle only once. For example, in Fig. 2, although $\Delta \left( 1,2,3\right)$ is a type-1 triangle that exists in $G_{12}$, $G_{13}$, and $G_{14}$, the algorithm considers it only in $G_{12}$ only. Also, $\Delta \left( 10,11,12\right)$ is a type-1 triangle where the partition number of its three nodes is $\rho$ in which it exists in $G_{14}$, $G_{24}$, and $G_{34}$. The algorithm identifies it only in the last sub-graph $G_{34}$. If *w*’s id is between *u* and *v* (i.e. $w\prec u,v$) (Line 23) and the triangle is not Type-1 (Line 28), then it counts this triangle (Line 30). For example, in Fig. 2, if the input of reduce instance is $\prec \left( 1,2\right) ; \{\left( 1,2\right) ,\left( 1,3\right) , \left( 2,3\right) ,\left( 2,4\right) ,\left( 3,4\right) , \left( 4,5\right) ,\left( 5,6\right) \}\succ$, the output will be $\prec \left( 1,2,3\right) ; 1 \succ$ (i.e. Type-1 triangle), and $\prec \left( 2,3,4\right) ; 1\succ$ (i.e. Type-2 triangle); if the input of reduce instance is $\prec \left( 1,2,4\right) ; \{\left( 2,4\right) ,\left( 3,4\right) ,\left( 3,10\right) , \left( 4,10\right) \}\succ$, the output will be $\prec \left( 3,4,10\right) ; 1\succ$ (i.e. Type-3 triangle).

#### Analysis

##### **Lemma 5**

*Each triangle in the graph is counted exactly once by ETTP.*

##### *Proof*

Each Type-1 triangle, $\Delta \left( u,v,w\right)$, appears in 2-partition graph. So, Type-1 triangle is counted only once in the first sub-graph $G_{ij}$ it belongs, [$j=i+1$ and $i=P\left( u\right)$] or in the last sub-graph when the partition number of three nodes of the triangle belongs to the last partition [$j=i+1$ and $P\left( u\right) =\rho$]. So those two conditions allow Type-1 triangles to count only once. While, each one of Type-2 triangle, $\Delta \left( u,v,w\right)$, appears only once in 2-partition, $G_{P\left( u\right) P\left( w\right) }$, where $P\left( u\right) <P\left( w\right)$. Moreover, Type-2 triangle appears only in 2-partition sub-graph not 3-partition sub-graph because there is an inner-edge in the triangle of Type-2 that exists only in 2-partition sub-graph. Therefore, Type-2 is counted correctly. On the other hand, Type-3 triangles appear only once in 3-partition sub-graphs. Therefore, ETTP counts the triangles correctly and only once. $\square$

##### **Lemma 6**

*Expected number of all map instances output of ETTP is* $m\left( \rho -1\right) =O\left( m\rho \right)$.

##### *Proof*

The proof consists of two consequent steps. First, if map instance input is an inner-edge $\left( u,v\right)$, then the output is $G_{ij}$ where $i,j\in \left[ 1,\rho \right] , i\ne j$, and partition number of two nodes belongs to *i* or *j*. Therefore, the output of every inner-edge is $\rho -1$ time. The probability that an edge is inner-edge is $\frac{1}{\rho }$. So, probability of all inner-edge in the graph is $\frac{m}{\rho }$. Therefore, the expect size of inner-edges output is:

6 $$\begin{aligned} \frac{m}{\rho }\times \left( \rho -1\right) \end{aligned}$$

Second, if map instance input is cross-edge, then the output is both $G_{ij}$ and $G_{ijk}$ where $i,j,k\in \left[ 1,\rho \right]$ and $i\ne j\ne k$. Then, the output of every cross-edge for $G_{ij}$ is generated one time and the output of every cross-edge for $G_{ijk}$ is $\left( \rho -2\right)$ times. So, total output of every cross-edge is $1+\left( \rho -2\right) =\rho -1$ times. The probability of cross-edge is $\left( 1-\frac{1}{\rho }\right) m =\frac{\left( \rho -1\right) }{\rho }m$. Therefore, the expected number of cross-edges output is:

7 $$\begin{aligned} \frac{\left( \rho -1\right) }{\rho }m\times \left( \rho -1\right) =\frac{\left( \rho -1\right) ^2}{\rho }m \end{aligned}$$

From the above two steps, we include that the expected number of all map instances output of ETTP is:

8 $$\begin{aligned} \frac{m}{\rho }\left( \rho -1\right) +\frac{\left( \rho -1\right) ^2}{\rho }m&=m\left( \rho -1\right) \left( \frac{1}{\rho }-\frac{\rho -1}{\rho }\right) \nonumber \\&=m\left( \rho -1\right) =O\left( m\rho \right) \end{aligned}$$

$$\square $$

##### **Lemma 7**

*Expected number of each reduce instance input is* $O\left( \frac{m}{\rho ^2}\right)$.

##### *Proof*

Reduce instance input is $\prec \left( i,j\right) ; E_{ij}\succ$ or $\prec \left( i,j,k\right) ; E_{ijk}\succ$. The probability that two nodes of the edge are in a specific partition is $\frac{1}{\rho }\times \frac{1}{\rho }=\frac{1}{\rho ^2}$. For the 2-partition, it contains inner-edges and cross-edges of the graph. Since the expected number of two nodes of inner-edges in 2-partition equals $\frac{1}{\rho ^2}+\frac{1}{\rho ^2} =\frac{2}{\rho ^2}$, and the expected number of two nodes of cross-edges in the same partition equals $\frac{1}{\rho ^2}$. Hence, for m edges, the expected number of two nodes of inner-edges in the same partition equals $m\times \frac{2}{\rho ^2}=\frac{2m}{\rho ^2}$, and the expected number of two nodes of cross-edges in the same partition equals $m\times \frac{1}{\rho ^2}=\frac{m}{\rho ^2}$. Therefore, the expected number of edges in 2-partition equals:

9 $$\begin{aligned} \frac{2m}{\rho ^2}+\frac{m}{\rho ^2}=\frac{3m}{\rho ^2}=O\left( \frac{m}{\rho ^2}\right) \end{aligned}$$

For the 3-partition, it contains cross-edge only. The number of two nodes of the edge in 3- partition equals $$\left( {\begin{array}{c}3\\ 2\end{array}}\right) $$. Hence, the expected number of edges in 3-partition equals:

10 $$\begin{aligned} \left( {\begin{array}{c}3\\ 2\end{array}}\right) \times \frac{m}{\rho ^2}=\frac{3m}{\rho ^2}=O\left( \frac{m}{\rho ^2}\right) \end{aligned}$$

From the above two equations, we include that for any input, reduce instance takes $O\left( \frac{m}{\rho ^2}\right)$. $\square$

##### **Lemma 8**

*The running time of reduce instance of sparse graph is* $O\left( m\right)$.

##### *Proof*

It’s already proofed in *Lemma 4*. From *Lemma 7*, reduce instance takes $O\left( \frac{m}{\rho ^2}\right)$ as input, and assume graph is a sparse graph; Therefore, the running time of reduce instance is $O\left( \frac{m}{\rho ^2}\right)$. $\square$

##### **Theorem 2**

*The running time of reduce instance of ETTP algorithm is better than TTP algorithm*.

##### *Proof*

From *Lemma 8* (Assume graph is a sparse graph), the running time of ETTP algorithm is $O\left( \frac{m}{\rho ^2}\right)$. TTP algorithm also takes $O\left( \frac{m}{\rho ^2}\right)$ as input and running time of reduce instance is $O\left( m^{3/2}\right)$^9^; hence, the running time of reduce instance in TTP algorithm is $O\left( \left( \frac{m}{\rho ^2}\right) ^{3/2} \right)$. Therefore, the running time of reduce instance of ETTP algorithm is better than TTP algorithm. $\square$

## Experimental results

In this section, we present and discuss the experimental results of our algorithms. We ran our two algorithms on a set of datasets found in SNAP^24^ and compared their running time with TTP algorithm. The experiments are divided into two parts. In the first part, the three algorithms run locally on a single node running Hadoop and in the second part, the three algorithms run in a distributed made on a cluster of machines having Hadoop running on them. Table 2 shows the basic characteristic of the datasets used in the experiments.

**Table 2:** Characteristic of used datasets.

Dataset	Nodes	Edges	Triangles
wiki-Vote	7115	103689	608389
ego-Facebook	4039	88234	1612010
p2p-Gnutella08	6301	20777	2383
AS-733	6474	13895	6584
ca-AstroPh	18772	396160	1351441
ca-HepTh	9877	51971	28339
CA-HepPh	12008	237010	3358499
Brightkite_edges	58228	428156	494728
Email-Enron	36692	367662	727044
p2p-Gnutella31	62586	147892	2024
soc-Epinions	75879	508837	1624481
CA-CondMat	23133	186936	173361

### Single node

**Figure 8:**
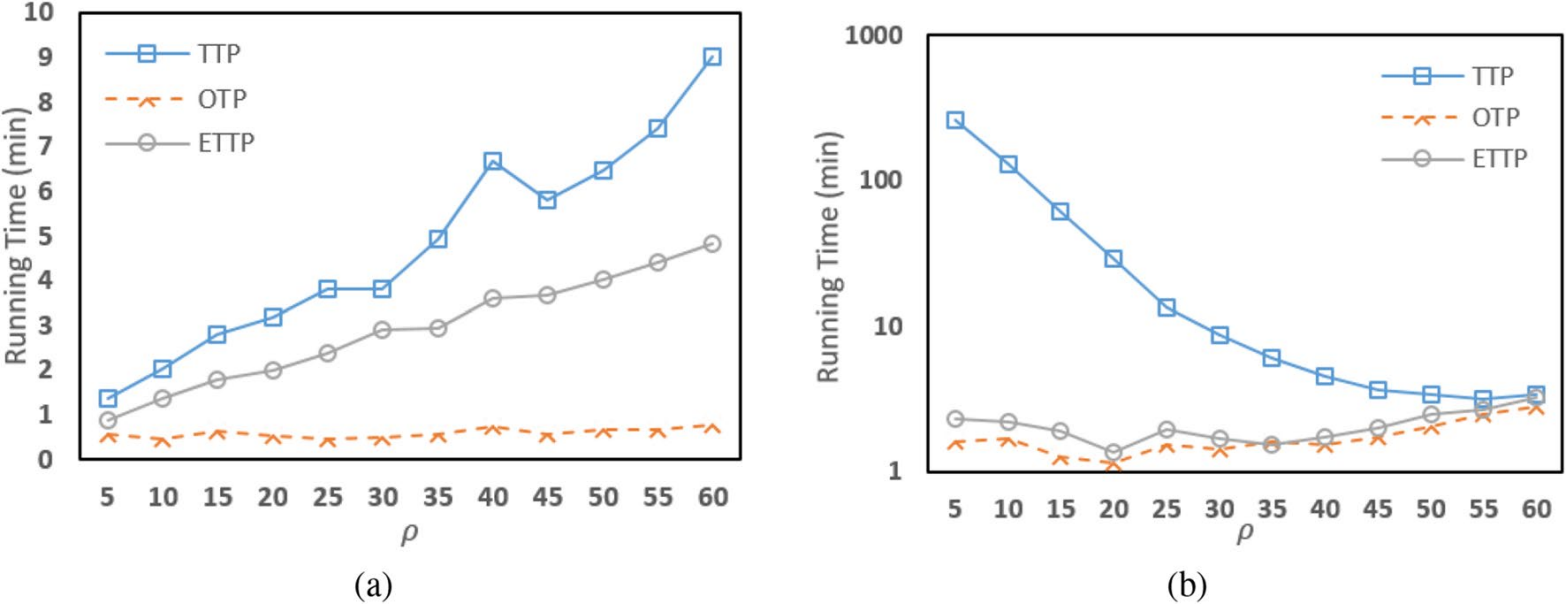
The running time of three algorithms in a single node with different $\rho$ size on: (**a**) ca-HepTh dataset, and (**b**) ego-Facebook dataset.

In the first set of experiments, the three algorithms are run on a single machine with Intel Core i5 processor, and 4GB RAM. This machine has Hadoop software running on it. Table 3 shows the running times of our two algorithms and TTP algorithm on this single node using a fixed number of partitions ($\rho =20$). From Table 3, we notice that our two algorithms, OTP and ETTP, always have running times smaller than that of TTP algorithm. In the case of big datasets with very high number of nodes and edges such as Brightkite_edges dataset, we notice that our two algorithms are much clearly faster than TTP algorithm, while OTP algorithm has better execution time than ETTP algorithm. Since, TTP algorithm takes 39.25 minutes, while ETTP algorithm takes 10.08 minutes, and OTP algorithm takes only 9.1 minutes. As can also be seen in the CA-CondMat dataset, OTP algorithm has a better performance time than ETTP algorithm which improved by almost 11 minutes. Hence; as expected, our two algorithms show remarkable improvement in running time as the OTP algorithm almost outperformed the ETTP algorithm. Moreover, to demonstrate the robustness of the proposed algorithms compared to TTP algorithm, we study the effect of different $\rho$ values on the three algorithms on ca-HepTh dataset and ego-Facebook dataset as shown in Fig. 8. From Fig. 8, we notice that the running times of TTP and ETTP algorithms change when $\rho$ changes while the running time of OTP is nearly constant. Moreover, the running time of OTP algorithm outperformed ETTP and TTP algorithms using different $\rho$ values while ETTP algorithm is better than TTP algorithm. Thus, it can be concluded that OTP gives a better result than ETTP and TTP algorithms when applied on bigger datasets running on a smaller cluster.

**Table 3:** Running times of all algorithms in a single node in Hadoop (min).

Dataset	TTP	OTP	ETTP
wiki-Vote	2.3	1.43	1.47
ego-Facebook	29.18	1.15	1.37
p2p-Gnutella08	0.32	0.3	0.3
AS-733	0.42	0.37	0.37
ca-AstroPh	5.1	4.82	3.87
ca-HepTh	3.17	0.53	2
CA-HepPh	3.07	2.27	1.92
Brightkite_edges	39.25	9.1	10.08
Email-Enron	172.8	8.97	12.6
p2p-Gnutella31	3.45	2.27	2.97
CA-CondMat	54.05	2.87	14.33

### Multi node

**Figure 9:**
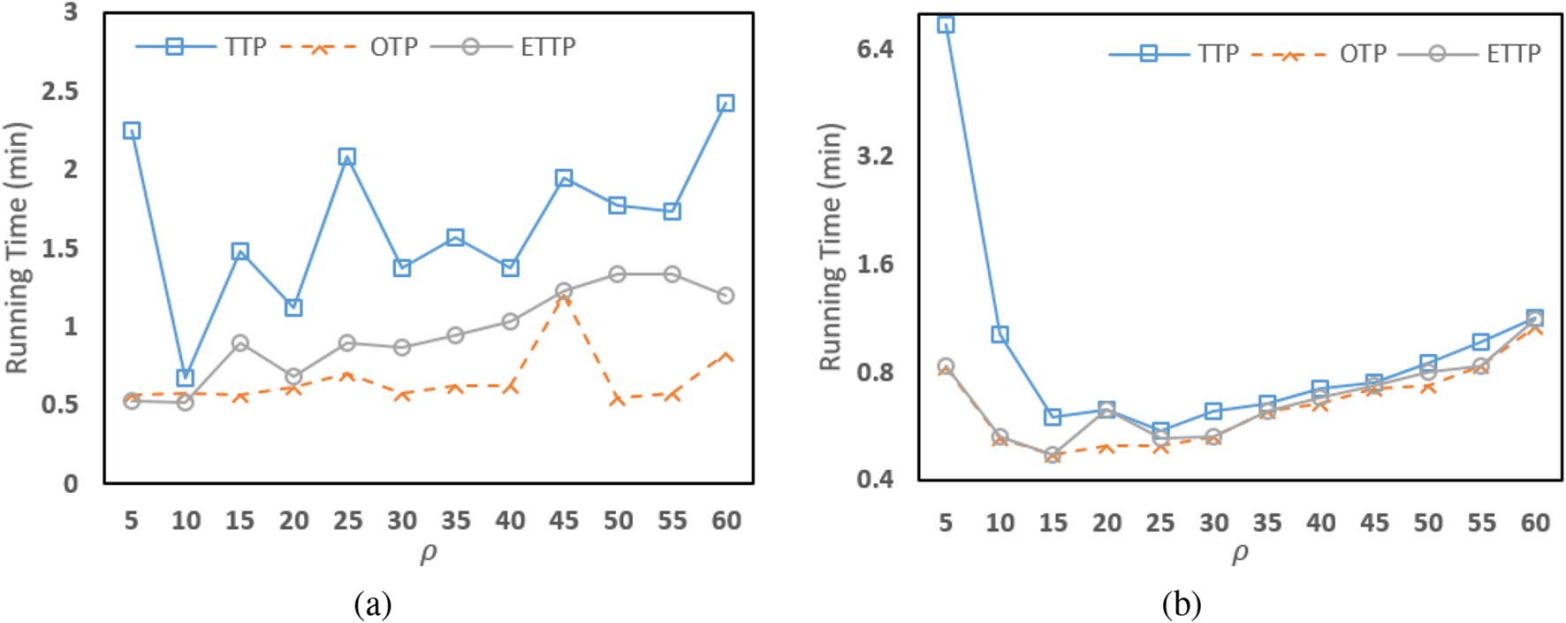
The running time of three algorithms in a multi-node with different $\rho$ size on: (**a**) ca-HepTh dataset, and (**b**) wiki-Vote dataset.

In the second set of experiments, the three algorithms are run on a cluster of 15 nodes (one master node and 14 slaves) running Hadoop framework. The 15 nodes are homogeneous and each node is a machine with Intel Core Quad processor, and 3.7 GB RAM. We run our two algorithms on the cluster and compare the results with TTP algorithm as shown in Table 4 with $\rho =20$. From Table 4, we notice that both our two algorithms are better than TTP algorithm. In the case of big dataset such as soc-Epinions dataset shown in Table 4, we notice that our two algorithms are much faster than TTP algorithm, and ETTP algorithm has better performance time than OTP algorithm. Therefore, our experimental results show that our two algorithms are faster than TTP algorithm, and OTP algorithm has better performance time than ETTP algorithm in smaller cluster. Also, we study the effect of number of partitions on the running times of the three algorithms applied in ca-HepTh dataset and wiki-Vote dataset as shown in Fig. 9. The figure shows that OTP and ETTP is more efficient than TTP algorithm when applied with different $\rho$ partitions.

**Table 4:** Running times of all algorithms in a multi node in Hadoop (min).

Dataset	TTP	OTP	ETTP
wiki-Vote	0.63	0.5	0.63
ego-Facebook	8.03	0.47	0.53
p2p-Gnutella08	0.2	0.2	0.18
AS-733	0.5	0.32	0.38
ca-AstroPh	1.23	1.15	1
ca-HepTh	1.12	0.62	0.68
CA-HepPh	0.82	0.72	0.7
Brightkite_edges	7.38	2.77	1.8
Email-Enron	23.58	3.22	1.97
p2p-Gnutella31	0.78	0.62	0.62
soc-Epinions	282.15	12.25	8.22
CA-CondMat	6.68	2.17	2.77

**Figure 10:**
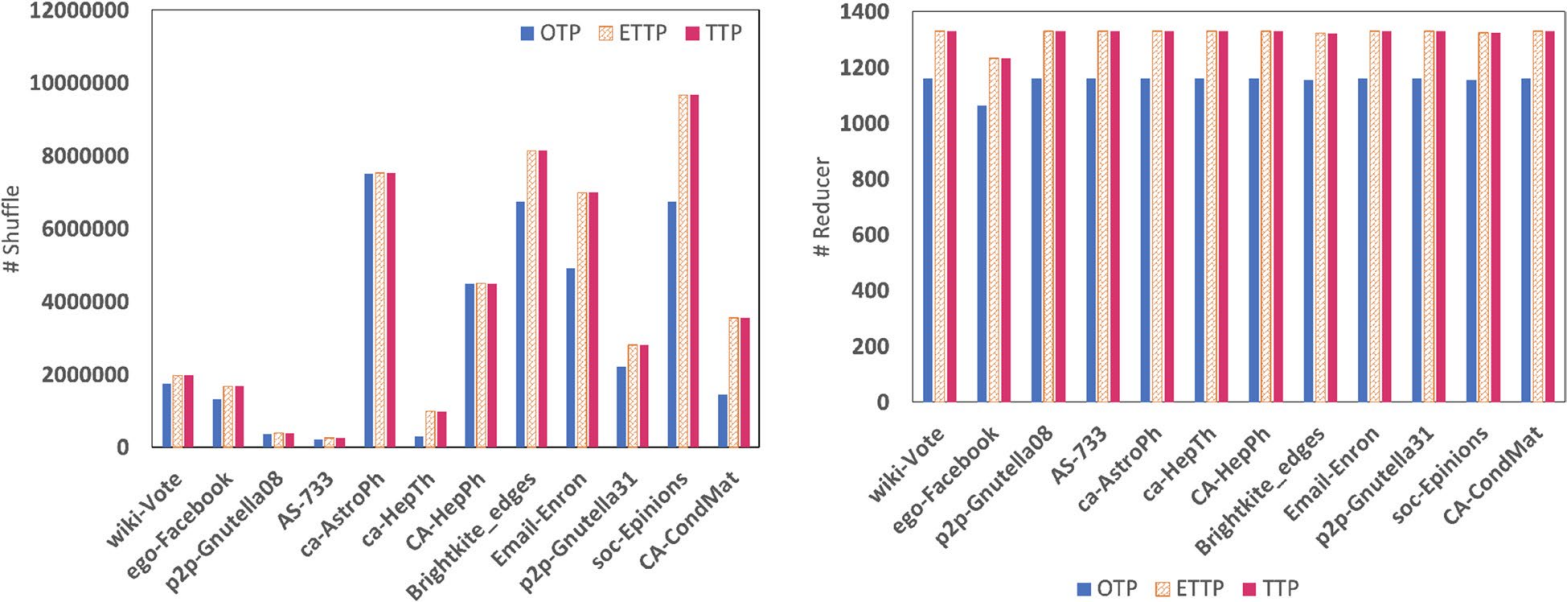
The workload of OTP, ETTP, and TTP algorithms.

Finally, we evaluate the workload of OTP, ETTP, and TTP as well in terms of the number of shuffles and the number of reducers as shown in Fig. 10. The figure shows that OTP has less workload than both ETTP and TTP. However, ETTP is better, as concluded earlier, and recommend to use in a large cluster of machines.

## Conclusion

Triangle counting is used significantly in many applications especially in social network analytics. Many researchers presented algorithms to solve this problem, but those algorithms can’t solve the problem properly due to the huge data. So, researchers use parallel algorithms over distributed frameworks (e.g. Hadoop MapReduce) to solve the problem as it is hard to use sequential algorithms to solve the problem. We use parallel algorithms to solve the problem, where we proposed two algorithms based on MapReduce parallel computing and graph partitioning to significantly enhance the time performance. The two proposed algorithms, ETTP and OTP, avoid repeated triangle counting by identifying each triangle only once in the graph. The experimental results show that ETTP and OTP algorithms give better execution time than the previous MapReduce algorithms, where ETTP is much better and recommended over OTP algorithm in a large cluster of machines. In the future, we plan to improve the performance of the proposed algorithms as well as evaluating the proposed algorithms on large datasets.

## Data Availability

The datasets generated and/or analysed during the current study are available in http://snap.stanford.edu/data/, accessed date: 9 April 2022.
